# Reliability and Accuracy of YouTube Peri-Implantitis Videos as an Educational Source for Patients in Population-Based Prevention Strategies

**DOI:** 10.3390/healthcare11142094

**Published:** 2023-07-23

**Authors:** Federica Di Spirito, Francesco Giordano, Maria Pia Di Palo, Davide Cannatà, Marco Orio, Nicoletta Coppola, Rossella Santoro

**Affiliations:** 1 Department of Medicine, Surgery and Dentistry, University of Salerno, 84081 Baronissi, Italy; frgiordano@unisa.it (F.G.); mariapia140497@gmail.com (M.P.D.P.); davide2897@icloud.com (D.C.); marcoorio818@gmail.com (M.O.); nicolettacoppola95@gmail.com (N.C.); 2 Multidisciplinary Department of Medical-Surgical and Odontostomatological Specialities, University of Campania “Luigi Vanvitelli”, 80138 Naples, Italy; rossella.santoro@unicampania.it

**Keywords:** peri-implantitis, peri-implant disease, prevention, preventive, education, educational, video, content, YouTube

## Abstract

Considering the need to improve patient knowledge, awareness, and compliance for peri-implantitis prevention, and patients’ demand for better, quick, and convenient access to medical information, the present study primarily assessed the reliability and accuracy of YouTube videos on peri-implantitis and secondarily evaluated their educational value for the patients, and the related suitability, as part of population-based preventive strategies, to deliver valid information, potentially capable of improving patient knowledge and educational skills. This study’s protocol was developed in advance, and computer history and cookies were cleared to avoid limitations based on preferred user histories. The search term “peri-implantitis” was defined using the Google Trends website, and videos were searched on YouTube on 5 December 2022. Video inclusion and data collection were conducted by independent pre-calibrated investigators. Descriptive statistics were performed on the videos’ characteristics, source, category, target audience, popularity, source reliability, video information and quality (VIQI), content, and educational value. Pearson’s correlation between educational value and all parameters was calculated. Videos with very low/low and medium/good/excellent educational value were compared using the Mann–Whitney U test. A total of 44 videos with medium values for popularity, VIQI, content, and educational value were analyzed. Most videos covered peri-implantitis treatment rather than etiology and prevention, about half were uploaded by dentists/specialists, and only 10% specifically targeted patients. Only 2% of YouTube videos about peri-implantitis had excellent educational value, and 5% had good educational value. Video educational value was correlated with VIQI and content beyond video length and source reliability. When comparing the very low/low and medium/good/excellent educational value YouTube videos on peri-implantitis, a significant difference was found in the Video Information and Quality Index, VIQI, and video content.

## 1. Introduction

Peri-implantitis is a destructive inflammatory process that affects the tissues surrounding dental implants and is characterized by mucosal inflammation and bone loss [1,2].

The prevalence of peri-implantitis ranges from 1% to 85% when considered at the patient rather than implant level [3], and the incidence rate increases from approximately 0.5% to 40% within 3 to 5 years after implant placement [3]. Periodontitis, smoking, and a lack of preventive interventions are currently recognized as risk factors for peri-implantitis [4], as are diabetes mellitus [4] and poor oral hygiene [5], whereas age, gender, and treated jaw did not appear to influence the incidence of peri-implantitis significantly [6]. The mean prevalence estimates of peri-implantitis were higher in periodontal subjects (9.8%) and patients who did not undergo regular prophylaxis (18.8%) than in the general population (7%) [4]. Overall, these findings support the protective role of controlling, or at least minimizing, modifiable and primarily behavioral risk factors among preventive interventions for peri-implantitis and reinforce the need for comprehensive, long-term population-based preventive measures [6].

Strategies to prevent peri-implantitis include routine clinical assessment and screening of peri-implant soft and hard tissues, periodontal maintenance care [7], and patient education and motivation [8].

Patient education and motivation, as well as treatment compliance, rely on effective communication with healthcare professionals [8]. For communication to be effective, information must be understood and remembered and can be transmitted to patients in many ways [8]. In the era of digitalization, sharing platforms, also known as social media, are currently considered the easiest and fastest way to share and receive information on any topic [9]. The ability of social media (such as YouTube, Instagram, Facebook, SnapChat, or m-health applications) to overcome physical barriers and the need for access to dental care has led to their increasing use for public oral health promotion [10]. Educating patients about home care through telehealth using downloadable medical applications on cell phones, called m-health applications, which include educational videos, has had good results, especially with regard to technologies for maintaining proper oral hygiene [11]. Likewise, YouTube videos with instructions on oral health, advice on food choices, fluoride use, and videos demonstrating proper tooth brushing have also shown educational value for children and parents [12]. Thus, these platforms are also known to improve the patients’ knowledge of health-related topics and increasingly influence opinion formation, including medical and dental content [13].

Several studies have been conducted to evaluate the impact of social media on oral health literacy, e.g., including fixed orthodontic treatment [8], orthodontic retention [14], lingual orthodontic treatment [15], space maintainers [16], pediatric oral health instructions [12], oral cancer [17], HPV-related oral lesions [18], burning mouth syndrome [19], Sjögren’s syndrome [20], stainless steel crowns [21], and complete arch fixed implant-supported prostheses [22]. These studies mainly focus on YouTube as a source of information, which is more likely to be used by patients compared to other websites, as the rich visual content and visual information is generally found to be much more appealing than readable materials [16].

Moreover, YouTube allows people to broadly network, collaborate, and share knowledge and experiences [23,24]. The latter is a key feature of the platform, as patients always seek interaction with a community of people with similar problems to share clinical information and experiences and receive support [25,26].

Furthermore, social media is able to combine the credibility of interpersonal persuasion with the echo chamber effect of mass media, resulting in desirable behavior among a large group of people [27,28], and different interfaces of several social media can generate diverse responses in the users [29].

In turn, however, any YouTube user can upload content, regardless of background and reliability. As a result, the quality of information is not guaranteed, and inaccurate content may mislead patients, negatively affecting preventive as well as therapeutic interventions [16]. For this reason, the information provided to patients should be evaluated with the available tools according to the type of communication [30].

Therefore, this study was conducted considering the need to improve patient knowledge, awareness, and compliance for peri-implantitis prevention [31,32,33].

## 2. Materials and Methods

### 2.1. Study Design

The present cross-sectional evaluation of Internet-based video media did not require approval from the Local Research Ethics Committee, as it contains only public data.

This study’s protocol was developed before the search. Before searching, the computer history and cookies were deleted to prevent restrictions based on preferential user history.

### 2.2. Search Strategy

YouTube (freely available at http://www.youtube.com) (accessed on 5 December 2022) was searched for relevant videos uploaded until 5 December 2022. The search keywords “peri-implantitis treatment” were defined through the Google Trends Web site (freely available at https://trends.google.com/trends/), with “worldwide” and “last 5 years” settings, and no related queries available. The filters applied to the YouTube search were the following: video duration between 4 and 20 min, ranking based on the “level of relevance”.

Since previous studies have shown that 80–90% [14] of the search results may change on different days, all the videos’ source locations (URL) were backed up and recorded.

### 2.3. Eligibility Criteria

Two independent investigators (M.P.D.P., D.C.) with experience in oral medicine and oral surgery independently screened eligible videos. Multipart videos were considered single videos.

The inclusion criteria were as follows:

- Video quality: ≥240p.
- Video language: English only.
- Primary video content: peri-implantitis treatment.

The exclusion criteria were as follows:

- Video quality: <240p.
- Video language: non-English.
- Video duplication.
- Videos without sound or written explanation.
- Advertisements from YouTube.

### 2.4. Data Collection

The calibration of 10 videos randomly chosen was performed by three investigators (F.G., M.O., N.C.) with experience in oral medicine and oral surgery.

The following data were independently extracted, computed, and collected by the two investigators (M.O., N.C.) involved in the pre-calibration on a standardized extraction form for each video included in the present study:

▪ Characteristics: link, length of video (minutes); number of views; number of likes; number of dislikes; number of comments; number of subscriptions; time elapsed since upload.
▪ Source: classified as dentist/specialist; hospital/university/scientific dental associations; commercial; other.
▪ Category: education; people and blogs; science and technology; film and animation; others.
▪ Target audience: classified as laypersons; professional; both.
▪ Video Power Index (VPI) [9,21].
▪ Video Information and Quality Index (VIQI) [21].
▪ Video content [22].
▪ Video source reliability [34].
▪ Video educational value (GQS) [22,35,36].

### 2.5. Video Power Index

The Video Power Index, assessing video popularity, was computed as follows: Like ratio (Number of likes + Number of dislikes/Number of views × 100) × View ratio (Number of views/Number of days since the video was uploaded × 100)/100 [9,16,21].

### 2.6. Video Information and Quality Assessment

The video information and quality index (VIQI) examined the videos’ general quality, scoring 1–20 [35].

A five-point Likert-type scale (from 1 = low quality to 5 = high quality) was employed to evaluate each of the following criteria:

❖ “Flow of the information.
❖ Accuracy of the information.
❖ Quality (use of photographs, animation, reports from members of the public, video headings, and summary).
❖ Sensitivity (the consistency level between the video title and the content)”.

### 2.7. Video Content Assessment

The content of the videos was evaluated, providing a total content score of 1–5, according to the coverage of the following topics:

❖ Definition/diagnostic criteria.
❖ Etiology.
❖ Diagnosis.
❖ Prevention.
❖ Treatment (any).

### 2.8. Video Source Reliability

The reliability of the source of the medical information retrieved through videos was determined using four criteria, known as the Journal of American Medical Association (JAMA) benchmark criteria, suggested by Silberg et al. [33,34]

❖ “Authorship (authors and contributors, their affiliations, and relevant credentials should be provided)
❖ Attribution (references and sources for all content should be listed clearly, and all
❖ relevant copyright information reported)
❖ Disclosure (website “ownership” should be prominently and fully disclosed, as should any sponsorship, advertising, underwriting, commercial funding arrangements or support, or potential conflicts of interest)
❖ Currency (dates that content was posted and updated should be indicated)”.

### 2.9. Video Educational Value

The videos’ educational value was rated based on the five-point Global Quality Scale (GQS) criteria [22,35,36]:

❖ Score 1 = Poor quality; very unlikely to be of any use to patients.
❖ Score 2 = Poor quality but some information present; of very limited use to patients.
❖ Score 3 = Suboptimal flow, some information covered but important topics missing; somewhat useful to patients.
❖ Score 4 = Good quality and flow, most important topics covered; useful to patients.
❖ Score 5 = Excellent quality and flow; highly useful to patients.

Videos rated <3 were classified as having very low/low educational value, and those rated ≥3 as having medium/good/excellent educational value for patients’ education, as part of population-based preventive strategies.

### 2.10. Statistical Analysis

The normality of the data distribution was determined through the Shapiro–Wilk test (*p* value < 0.001). The descriptive statistical analysis was performed for all of the YouTube videos included. The correlation between the educational value and videos’ characteristics, popularity, Information and Quality Index (VIQI), content topics and score, and the video source reliability was computed through Pearson’s correlation test.

The included videos were categorized based on the educational values as very low/low (<3) and medium/good/excellent (≥3) educational value videos, analyzed, and compared with the Mann–Whitney U-test.

## 3. Results

### 3.1. YouTube Videos on Peri-Implantitis: Inclusion and Data Collection

Out of the 120 videos initially retrieved, a total of 44 YouTube videos on peri-implantitis compliant with the eligibility criteria were considered in the present study, mainly due to the inconsistency between the title and content and the lack of information provided on peri-implantitis.

Related data extracted, computed, and collected are available as a Supplementary Materials.

### 3.2. YouTube Videos on Peri-Implantitis: Descriptive Analysis

The median length of the videos was 5.58 (4.02–19.3) mins. The videos were uploaded between 27 and 2842 days (median time elapsed since upload 974) before the search and received a median number of views of 684, with a median number of likes of 6.5 (0–333), no dislikes, and a Video Power Index VPI of 1.09.

The median Video Information and Quality Index VIQI (0–20 score) was 12.0.

The median video educational value (0–5 score) was 2.0; thus, on average, the videos included were considered to be of low to medium educational value.

The included videos’ length, number of views, likes, dislikes, comments and subscriptions, the time elapsed since upload, the Video Power Index (VPI), the video source reliability, the Video Information and Quality Index (VIQI), the video content, and the video educational value (GQS) are detailed in Table 1 and Table 2.

The median video source reliability (0–4 score) was 2.5 and dentists/specialists uploaded 53.2% of the videos. Approximately 40% of the videos fell into the “education” category. About 10.6% of the videos were produced for laypersons.

**Figure 1:**
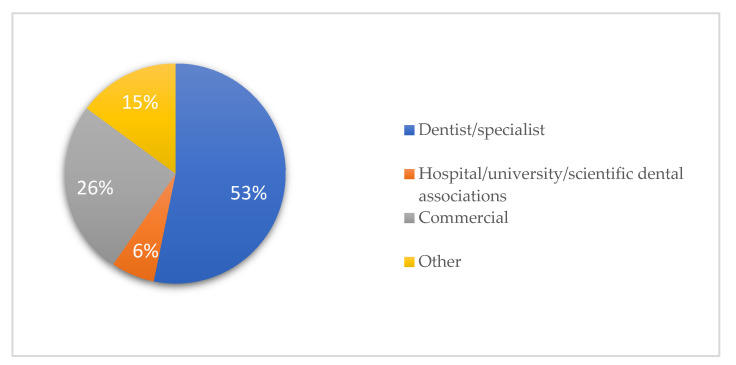
Sources of YouTube videos on peri-implantitis.

**Figure 2:**
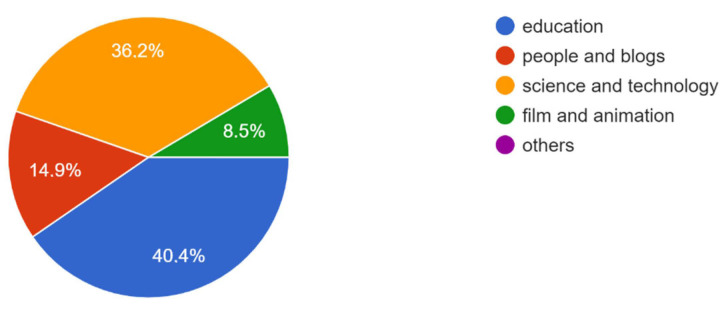
Categories of YouTube videos on peri-implantitis.

**Figure 3:**
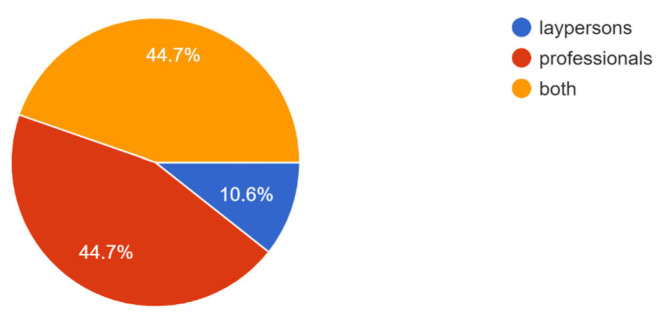
Target audience of YouTube videos on peri-implantitis.

The source, category, and target audience of YouTube videos on peri-implantitis are shown in Figure 1, Figure 2 and Figure 3.

The median video content score (1–5 score) was 2, indicating that, on average, YouTube videos covered at least two of the five relevant topics (definition/diagnostic criteria, etiology, diagnosis, prevention, treatment).

Peri-implantitis treatment was the most frequently covered topic (88.63%), followed by definition/diagnostic criteria (47.72%), etiology (36.33%), prevention (31.81%), and diagnosis (29.54%).

**Figure 4:**
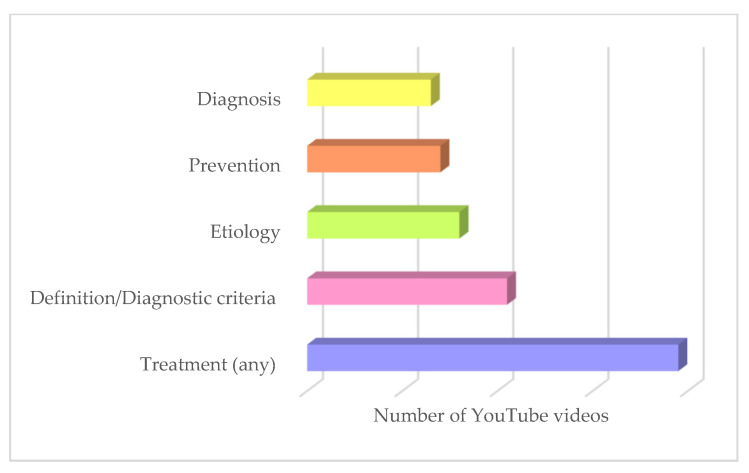
Content topics of YouTube videos on peri-implantitis.

The content topics of the videos are shown in Figure 4.

The educational value of the videos assessed using the five-point Global Quality Scale (GQS) criteria was distributed as follows: 7% of the videos had very low and 43% low value, whereas medium, good and excellent value was assigned to 34%, 11%, and 5% of the videos analyzed, respectively.

### 3.3. YouTube Videos on Peri-Implantitis: Correlation between Video Educational Value and Other Parameters

Pearson’s correlation coefficients measuring the strength of the linear association between the videos’ educational value and the videos’ characteristics of popularity (VPI), general quality (VIQI), content, and source reliability are listed in Table 3.

Video educational value was significantly related to the length of the video (r = 0.353, *p*-value = 0.016), the number of likes (r = 0.386, *p*-value = 0.008) and video source reliability (r = 0.314, *p*-value = 0.034), but not specifically related to the uploading dentist/specialist (r = 0.153, *p*-value = 0.311).

A highly significant correlation was found among the video educational value, the Video Information and Quality Index (r = 0.714, *p*-value < 0.001) and the video content score (r = 0.670, *p*-value < 0.001); specifically, the video educational value was significantly correlated with the “Definition/Diagnostic criteria” (r = 0.437, *p*-value < 0.002) and the “Diagnosis” (r = 0.529, *p*-value < 0.001) content topics, but not with “Prevention” and “Etiology”, nor with “Treatment” ones.

### 3.4. YouTube Videos on Peri-Implantitis: Comparison between Very Low/Low and Medium/Good/Excellent Educational Value Videos

A total of 22 YouTube videos on peri-implantitis were rated <3 and classified as having very low/low educational value, and the remaining 22 were rated ≥3 and classified as having medium/good/excellent educational value, based on the five-point Global Quality Scale (GQS).

Variables of very low/low and medium/good/excellent educational value videos are reported in Table 4.

When comparing the very low/low and medium/good/excellent educational value YouTube videos on peri-implantitis, a significant difference was found in the Video Information and Quality Index (*p*-value < 0.001) and video content (*p*-value < 0.001).

The comparison of variables between very low/low and medium/good/excellent educational value videos computed with the Mann–Whitney U test is shown in Table 5.

## 4. Discussion

### 4.1. YouTube Videos on Peri-Implantitis: Reliability and Accuracy

A total of 44 YouTube videos on peri-implantitis were included in the present cross-sectional analysis of educational reliability and accuracy to improve patient knowledge, awareness, and compliance as a population-based prevention strategy (Table 1). Compared with the 120 videos initially found in the search, this reduced number of videos analyzed was mainly due to inconsistencies between the title and the content, and the lack of information provided on peri-implantitis. Limiting the videos’ length to 4–20 min certainly reduced the number of search results, but based on the suggested optimal video duration to maintain viewer attention, ranging from 5–6 [37,38] to 10 min [37,38], indirectly increased the likelihood that YouTube videos were viewed in their entirety, making the presented results generalizable, particularly with regard to population-based prevention strategies, although the median length of the 44 videos was 5.58 min. However, data on viewing duration could not be retrieved from YouTube.

The total number of views of the videos analyzed varied widely (median = 684.00) (Table 2), suggesting a limited distribution of the videos.

The popularity of the videos, although no dislikes were recorded, was even more modest, with a median number of likes of 6.50 and a median Video Power Index of 1.09 (Table 2), suggesting the videos’ low appeal. In any case, it should be noted that the time elapsed from video upload ranged from 27 days to approximately 6 years (Table 2), with a median of 974 days (about 3 years), revealing a relatively recent introduction of peri-implantitis content to YouTube.

Less than half (40.4%) of the YouTube videos on peri-implantitis were in the “education” category (Figure 3), and only 10.6% were directed at laypersons (Figure 2), indicating that most of the videos were probably not uploaded for educational purposes for patients. Accordingly, the median video content score (1–5 score) was 2.0 (Table 1), indicating that these YouTube videos, on average, covered at least two of the five topics examined (definition/diagnostic criteria, etiology, diagnosis, prevention, treatment), and thus offered to patients incomplete information about peri-implantitis, even when considering multi-part videos as a whole.

Moreover, the most frequently covered content was the treatment of peri-implantitis (88.63%), followed by definition/diagnostic criteria (47.72%), and less so by etiology (36.33%) and prevention (31.81%) (Figure 4), which is critical for patient knowledge and awareness regarding the control of peri-implantitis risk factors.

Furthermore, 53.2% of the videos were uploaded by dentists/specialists, while only 6.4% were uploaded by recognized institutions, such as universities or hospitals (Figure 1), with a median video source reliability (0–4 score) of 2.50 (Table 1). Notably, YouTube videos on peri-implantitis were the least likely to meet the “disclosure” criterion, similar to peri-implantitis websites [39].

Monje et al. [32] advocated the use of visual aids in combination with essential and clearly presented information to better inform patients and induce positive behavior changes. YouTube, as an online video-sharing and social media platform, can overcome the critical problem of search engines such as Google^®^ and Yahoo!^®^ by providing easier-to-understand visual content to improve patient knowledge and awareness of population-based prevention strategies, potentially playing a key role in conveying medical and dental information to patients and improving their understanding [39]. Conversely, patient-oriented online information about peri-implantitis on Internet websites, particularly Google^®^ and Yahoo!^®^ [39], was found to be challenging to understand due to complex and technical terminology and, consequently, of limited use for patient education. However, the median Video Information and Quality Index (0–20 score) was 12.00 (Table 1), reflecting how the flow and accuracy of the information, the consistency between the video title and content, previously discussed, and overall video quality (video headlines, summary, photos, animations, etc.) were generally moderate.

### 4.2. YouTube Videos on Peri-Implantitis: Educational Value

Unfortunately, half of the YouTube videos on peri-implantitis were of very low/low educational value (GQS < 3), and the other half were of medium/good/excellent educational value (GQS ≥ 3); specifically, 34.09% of the videos had a medium educational value, and only 11.36% and 4.54% had a good and excellent educational value, respectively. Thus, the majority of the YouTube videos on peri-implantitis analyzed were rated as poor for patient education.

These results are consistent with those of YouTube videos on other medical and dental content. Indeed, the information quality of YouTube videos on orthopedics [9] and allergology/immunology [40] has been rated as inadequate or misleading. Studies on the reliability of YouTube videos on the rehabilitation of complete dental arches with dental implants [16], endodontic treatment [41], and burning mouth syndrome [19] also reached similar conclusions. In contrast, two-thirds of YouTube videos on type 2 diabetes [42] [Leong, 2017] and more than half of the videos on Sjogren’s syndrome [20] were found to be educationally valid, with a good or excellent global quality rating.

Similar to the results reported by Lena et al. [15], who analyzed YouTube videos on lingual orthodontic treatment, the educational value of peri-implantitis videos was significantly related to video length (r = 0.353, *p*-value = 0.016) (Table 3), suggesting that conveying accurate and complete information requires appropriate time, as previously described [37,38]. However, when comparing duration, no statistical difference was found between high- and low-educational value videos on peri-implantitis (Table 5) as well as on stainless steel crowns [21].

In previous studies, videos with low information and quality and low educational value (low VIQI and GQS) were often more popular and preferred among YouTube users, probably because they were easier to understand for people without a medical background [9,19]. In contrast, in the present study, the educational value of the videos was significantly related to the number of likes (r = 0.386, *p*-value = 0.008) (Table 3). This is likely due to the fact that most YouTube videos about peri-implantitis targeted a professional audience (44.7%) (Figure 3), who find videos with high-quality and medium/good/excellent educational value the most useful to enrich their cultural background.

Although videos produced by healthcare professionals or institutions were found to be of greater educational value [43], a nonsignificant association was found between the uploaders and the educational value (Table 3). Conversely, as expected, the educational value of the videos was significantly associated with video source reliability (r = 0.314, *p*-value = 0.034) (Table 3).

When comparing YouTube videos on peri-implantitis with very low/low and medium/good/excellent educational value, a significant difference was found in the Video Information and Quality Index (*p*-value < 0.001) and video content score (*p*-value < 0.001) (Table 5), possibly illustrating how closely the accuracy and completeness of the information provided is related to its ability to convey valid information potentially capable of improving the patients’ knowledge and educational skills.

The main limitations of the present cross-sectional analysis of the reliability and accuracy of YouTube videos in improving patient knowledge, awareness, and compliance may be ascribable to the dynamic nature of the platform, where videos are uploaded and deleted daily, and the restriction of the language of the videos to English, considering that other languages, especially Chinese, Hindi, and Spanish, are even more widely used (data available free online at: https://en.wikipedia.org/wiki/List_of_languages_by_total_number_of_speakers) (accessed on 5 December 2022).

However, the present study may be the first to examine the popularity, information and quality, content, and source of YouTube videos on peri-implantitis and to evaluate the associated content accuracy, source reliability, and educational value. In addition, the results presented were compared with those of a recent study assessing the intelligibility of educational content on peri-implantitis websites to comprehensively assess the suitability of available patient-centered Internet information for population-based peri-implantitis prevention strategies [44].

Further cross-sectional analyzes should be conducted without language restrictions, including other social media platforms, and should be continuously updated. Indeed, dentists should be aware of the information available on the Internet and refer patients to appropriate sources with accurate and up-to-date content to improve the suitability of YouTube videos as an educational resource for patients in population-based prevention strategies against peri-implantitis.

Future studies of patient-centered, educational Internet information for peri-implantitis prevention should evaluate not only the accuracy of the information, but also its impact on patient knowledge, awareness, and compliance, as well as methods that are more engaging and effective for patients [45]. A pool of good/excellent educational videos on YouTube aimed at the general population and focusing primarily on recognized modifiable, particularly behavioral, risk factors associated with peri-implantitis and the benefits of preventive measures could be specifically developed.

In addition to videos aimed at a professional audience [46], patient-centered videos can be developed. They should be short (no longer than 10–15 min) in order to retain the viewers’ attention, have a cartoon-like design in order to be attractive, and present precise and synthetic information clearly, in order to avoid fatigue and distraction, in simple and essential language combined with visual aids to achieve better understanding [38,47].

## 5. Conclusions

Less than half of the YouTube videos on peri-implantitis fell into the “education” category, and the language used was often technical and difficult for patients to understand, making it unlikely that they were uploaded for patient education purposes. The educational value of the videos was mostly rated as poor and was significantly related to video length, source reliability, the Video Information and Quality Index, and video content.

Most of the videos covered the treatment of peri-implantitis rather than its prevention and etiology, which are critical for patient knowledge.

## Figures and Tables

**Table 1:** YouTube videos on peri-implantitis: general characteristics.

	Median	Range	Minimum	Maximum
Videos length (min)	5.58	15.2	4.02	19.3
Number of views	684.00	33,743.0	24.00	33,823.00
Number of likes	6.50	333	0	333
Number of dislikes	0.00	0.00	0	0
Number of comments	0.00	48	0	48
Number of subscriptions	1460.00	33,100	0	33,100
Time elapsed since upload (days)	974.00	2815	27	2842

**Table 2:** YouTube videos on peri-implantitis: Video Power Index “VPI”; Video Information and Quality Index “VIQI” (1–20 total score); video content on peri-implantitis (1–5 total score); video source reliability based on JAMA benchmark (0–4 total score); video educational value (0–5 total score).

	Median	Range	Minimum	Maximum
Video Power Index	1.09	181.3	0.00	181.3
Video Information and Quality Index (0–20 score)	12.00	16	4.00	20.00
Video content (1–5 score)	2.00	4	1.00	5.00
Video source reliability (0–4 score)	2.50	3	1.00	4.00
Video educational value (0–5 score)	2.00	4	1.00	5.00

**Table 3:** Correlation between the videos’ educational value and the videos’ characteristics, popularity (VPI), general quality (VIQI), content score and domains, source reliability, and dentist/specialist source was computed using Pearson’s correlation test.

	Educational Value of YouTube Videos on Peri-Implantitis	
Variables	Pearson’s r	*p*-Value
Length	0.353	0.016
Number of views	−0.078	0.605
Number of likes	0.386	0.008
Number of dislikes	0	0
Number of comments	0.191	0.203
Number of subscriptions	0.271	0.069
Time elapsed since upload (days)	−0.237	0.113
Video Power Index	0.153	0.311
Video Information and Quality Index	0.714	<0.001
Video content (1–5 score)	0.670	<0.001
Definition/Diagnostic criteria	0.437	0.002
Etiology	0.261	0.080
Diagnosis	0.529	<0.001
Prevention	0.179	0.235
Treatment (any)	0.083	0.584
Video source reliability	0.314	0.034
**Dentist/Specialist**	0.153	0.311

**Table 4:** Variables of very low/low and medium/good/excellent educational value YouTube videos on peri-implantitis.

YouTube Videos on Peri-Implantitis	Very Low/Low Educational Value			Medium/Good/Excellent Educational Value		
	Minimum	Median	Maximum	Minimum	Median	Maximum
Length (min)	4.02	5.48	15.3	4.13	8.105	19.3
Number of views	24.00	579.50	33,767	27.19	835.00	7706.0
Number of likes	0.00	3.50	272	0.00	14.50	333
Number of dislikes	0.00	0.00	0.000	0.00	0.00	0.000
Number of comments	0.00	0.00	48.00	0.00	0.50	27.00
Number of subscriptions	0.00	1335.00	33,100	0.00	6030.00	31,600
Time elapsed since upload (days)	158	1495.00	2842	27.00	802.50	2817
Video Power Index	0.00	0.325	16.90	0.00	3.60	181.3
Video Information and Quality Index	4.00	10.00	19.00	10.00	14.50	20.00
Video content	1.00	2.00	4.00	1.00	3.00	5.00
Definition/Diagnostic criteria	0.00	0.00	1.00	0.00	1.00	1.00
Etiology	0.00	0.00	1.00	0.00	0.00	1.00
Diagnosis	0.00	0.00	1.00	0.00	1.00	1.00
Prevention	0.00	0.00	1.00	0.00	0.00	1.00
Treatment (any)	0.00	1.00	1.00	0.00	1.00	1.00
Video source reliability (0–4 score)	1.00	2.00	4.00	1.00	3.00	4.00
Video educational value (0–5 score)	1.00	2.00	2.00	3.00	3.00	5.00

**Table 5:** Comparison of variables between very low/low and medium/good/excellent educational value YouTube videos on peri-implantitis.

Very Low/Low vs. Medium/Good/Excellent Educational Value YouTube Videos on Peri-Implantitis	Statistic ^1^	*p*-Value
Length of video	−1.282	0.206
Time elapsed since upload (days)	1.311	0.197
Video Power Index	−1.452	0.153
Video Information and Quality Index (0–20 score)	−4.304	<0.001 *
Video content (1–5 score)	−4.390	<0.001 *
Video source reliability based on the JAMA benchmark criteria (0–4 score)	−1.405	0.167
Video educational value based on the Global Quality Scale “GQS”	−8.139	<0.001

^1^ Mann–Whitney test; * Statistically significant.

## Data Availability

Data supporting reported results can be found in Web of Science, Scopus, and MEDLINE/PubMed databases.

## Supplementary Materials

The following supporting information can be downloaded at: https://www.mdpi.com/article/10.3390/healthcare11142094/s1, Table S1: Data extracted and computed from the videos included.
