# Supplementary material for: Reliability and Accuracy of YouTube Peri-Implantitis Videos as an Educational Source for Patients in Population-Based Prevention Strategies
Source: Healthcare (Basel). 2023 Jul 23;11(14):2094. doi: 10.3390/healthcare11142094 (PMC10378864; doi:10.3390/healthcare11142094)
Supplement: Supplementary file 1 [file healthcare-11-02094-s001.zip › healthcare-2463084-supplementary.pdf]

Table S1. Data extracted and computed from the videos included.
Characteristics: link; length (minutes); time elapsed since upload (days); target audience (layperson; professional; both).
Source and Category: source (dentist/specialist; hospital/university/scientific dental associations; commercial; other); category (education; people and blogs; science and technology; film and animation; others); Video Source Reliability based on JAMA benchmark (0-4 total score).
Popularity: views (n.); likes (n.); dislikes (n.); comments (n.); subscriptions (n.); like ratio; view ratio; Video Power Index "VPI".
Information and quality: information flow (1-5 score); information accuracy (1-5 score); quality (1-5 score); sensitivity (1-5 score); Video Information and Quality Index "VIQI" (1-20 total score).
Content on peri-implantitis: definition (yes=1/no=0); etiology (yes=1/no=0); diagnosis (yes=1/no=0); prevention (yes=1/no=0); treatment (yes=1/no=0); total content score (1-5).

Characteristics	Source and Category	Popularity	Information and Quality	Content	Educational Value
Link: https://www.youtube.com/watch?app=desktop&v=afOjNTOoPEE<br><br>Lenght (min):11:03<br><br>Time elapsed since upload (days): 2362<br><br>Target audience: Both	Source: Other<br><br>Category: Education<br><br>**Video Source Reliability (JAMA):** 1	Views (n.33767)<br>Likes (n.272)<br>Dislikes (n.0)<br>Comments (n.16)<br>Subscriptions (n.3450)<br><br>Like Ratio: 0,805<br>View Ratio: 1429,59<br><br>**VPI:** 11,5	Information flow (5)<br>Information accuracy (5)<br>Quality (4)<br>Sensitivity (5)<br><br>**VIQI:** 19	Definition (0)<br>Etiology (0)<br>Diagnosis (0)<br>Prevention (0)<br>Treatment (any) (1)<br><br>**Content score:** 1	**Educational value (GQS):** 2- Poor quality but some information present; of very limited use to patients

Link: https://www.youtube.com/watch?app=desktop&v=5bOjhHsCa_Y<br><br>Lenght (min):12:20<br><br>Time elapsed since upload: 809<br><br>Target audience: Both	Source: Other<br><br>Category: Education<br><br>**Video Source Reliability (JAMA):** 1	Views (n.4283)<br>Likes (n.151)<br>Dislikes (n.0)<br>Comments (n.8)<br>Subscriptions (n.22700)<br><br>Like Ratio: 3,53<br>View Ratio: 529,42<br><br>**VPI:** 18,69	Information flow (4)<br>Information accuracy (3)<br>Quality (1)<br>Sensitivity (3)<br><br>**VIQI:** 11	Definition (1)<br>Etiology (1)<br>Diagnosis (1)<br>Prevention (0)<br>Treatment (any) (1)<br><br>**Content score:** 4	**Educational value (GQS):** 3- Suboptimal flow, some information covered but important topics missing; somewhat useful to patients
Link: https://www.youtube.com/watch?app=desktop&v=uiu6GgB8bZk<br><br>Lenght (min):15:34<br><br>Time elapsed since upload: 158<br><br>Target audience: Professional	Source: Other<br><br>Category: Science and technology<br><br>**Video Source Reliability (JAMA):** 1	Views (n.594)<br>Likes (n.12)<br>Dislikes (n.0)<br>Comments (n.0)<br>Subscriptions (n.158)<br><br>Like Ratio: 2,02<br>View Ratio: 375,95<br><br>**VPI:** 7,59	Information flow (3)<br>Information accuracy (3)<br>Quality (1)<br>Sensitivity (2)<br><br>**VIQI:** 9	Definition (1)<br>Etiology (1)<br>Diagnosis (1)<br>Prevention (0)<br>Treatment (any) (1)<br><br>**Content score:** 4	**Educational value (GQS):** 2- Poor quality but some information present; of very limited use to patients

Link: https://www.youtube.com/watch?app=desktop&v=i0Gvr-lwxFc<br><br>Lenght (min):4:13<br><br>Time elapsed since upload: 1303<br><br>Target audience: Both	Source: Other<br><br>Category: Education<br><br>**Video Source Reliability (JAMA):** 3	Views (n.1607)<br>Likes (n.17)<br>Dislikes (n.0)<br>Comments (n.1)<br>Subscriptions (n.956)<br><br>Like Ratio: 1,06<br>View Ratio: 123,33<br><br>**VPI:** 1,31	Information flow (2)<br>Information accuracy (3)<br>Quality (3)<br>Sensitivity (5)<br><br>**VIQI:** 13	Definition (0)<br>Etiology (0)<br>Diagnosis (0)<br>Prevention (0)<br>Treatment (any) (1)<br><br>**Content score:** 1	**Educational value (GQS):** 3- Suboptimal flow, some information covered but important topics missing; somewhat useful to patients
Link: https://www.youtube.com/watch?app=desktop&v=M361YtdeoiM<br><br>Lenght (min):15:21<br><br>Time elapsed since upload: 764<br><br>Target audience: Professional	Source: Commercial<br><br>Category: Science and technology<br><br>**Video Source Reliability (JAMA):** 1	Views (n.1786)<br>Likes (n.38)<br>Dislikes (n.0)<br>Comments (n.0)<br>Subscriptions (n.6030)<br><br>Like Ratio: 2,13<br>View Ratio: 233,77<br><br>**VPI:** 4,98	Information flow (4)<br>Information accuracy (4)<br>Quality (4)<br>Sensitivity (5)<br><br>**VIQI:** 17	Definition (1)<br>Etiology (0)<br>Diagnosis (0)<br>Prevention (0)<br>Treatment (any) (1)<br><br>**Content score:** 2	**Educational value (GQS):** 3- Suboptimal flow, some information covered but important topics missing; somewhat useful to patients

Link: https://www.youtube.com/watch?app=desktop&v=uvb5udFZ3eA<br><br>Lenght (min):4:50<br><br>Time elapsed since upload: 932<br><br>Target audience: Professional	Source: Dentist/specialist<br><br>Category: Science and technology<br><br>**Video Source Reliability (JAMA):** 3	Views (n.1778)<br>Likes (n.53)<br>Dislikes (n.0)<br>Comments (n.4)<br>Subscriptions (n.6030)<br><br>Like Ratio: 2,99<br>View Ratio: 190,77<br><br>**VPI:** 5,70	Information flow (2)<br>Information accuracy (2)<br>Quality (2)<br>Sensitivity (4)<br><br>**VIQI:** 10	Definition (0)<br>Etiology (0)<br>Diagnosis (0)<br>Prevention (1)<br>Treatment (any) (0)<br><br>**Content score:** 1	**Educational value (GQS):** 2- Poor quality but some information present; of very limited use to patients
Link: https://www.youtube.com/watch?app=desktop&v=hiubSWcZLCM<br><br>Lenght (min):4:54<br><br>Time elapsed since upload: 970<br><br>Target audience: Both	Source: Other<br><br>Category: Education<br><br>**Video Source Reliability (JAMA):** 4	Views (n.3538)<br>Likes (n.35)<br>Dislikes (n.0)<br>Comments (n.1)<br>Subscriptions (n.7280)<br><br>Like Ratio: 0,99<br>View Ratio: 364,74<br><br>**VPI:** 3,61	Information flow (3)<br>Information accuracy (3)<br>Quality (2)<br>Sensitivity (2)<br><br>**VIQI:** 10	Definition (1)<br>Etiology (1)<br>Diagnosis (1)<br>Prevention (0)<br>Treatment (any) (0)<br><br>**Content score:** 3	**Educational value (GQS):** 3- Suboptimal flow, some information covered but important topics missing; somewhat useful to patients

Link: https://www.youtube.com/watch?app=desktop&v=2LHj_V0QjsA<br><br>Lenght (min):6:45<br><br>Time elapsed since upload: 1092<br><br>Target audience: Both	Source: Dentist/specialist<br><br>Category: Education<br><br>**Video Source Reliability (JAMA):** 3	Views (n.565)<br>Likes (n.16)<br>Dislikes (n.0)<br>Comments (n.0)<br>Subscriptions (n.1230)<br><br>Like Ratio: 2,83<br>View Ratio: 51,74<br><br>**VPI:** 1,46	Information flow (3)<br>Information accuracy (3)<br>Quality (4)<br>Sensitivity (2)<br><br>**VIQI:** 12	Definition (0)<br>Etiology (0)<br>Diagnosis (0)<br>Prevention (0)<br>Treatment (any) (1)<br><br>**Content score:** 1	**Educational value (GQS):** 2- Poor quality but some information present; of very limited use to patients
Link: https://www.youtube.com/watch?app=desktop&v=MhB_PlNM7CE<br><br>Lenght (min):4:02<br><br>Time elapsed since upload: 196<br><br>Target audience: Layperson	**Source and Category**<br><br>Source: Dentist/specialist<br><br>Category: People and blogs<br><br>**Video Source Reliability (JAMA):** 2	Views (n.88)<br>Likes (n.0)<br>Dislikes (n.0)<br>Comments (n.0)<br>Subscriptions (n.16)<br><br>Like Ratio: 0<br>View Ratio: 44,90<br><br>**VPI:** 0	Information flow (2)<br>Information accuracy (2)<br>Quality (2)<br>Sensitivity (3)<br><br>**VIQI:** 9	Definition (0)<br>Etiology (0)<br>Diagnosis (0)<br>Prevention (0)<br>Treatment (any) (1)<br><br>**Content score:** 1	**Educational value (GQS):** 2- Poor quality but some information present; of very limited use to patients

Link:<br>https://www.youtube.com/watch?app=desktop&v=8X0QY1tdPbg<br><br>Lenght (min):7:13<br><br>Time elapsed since upload: 2817<br><br>Target audience:<br>Both	Source:<br>Dentist/specialist<br><br>Category:<br>Science and technology<br><br>**Video Source Reliability (JAMA):** 1	Views (n.7706)<br>Likes (n.7)<br>Dislikes (n.0)<br>Comments (n.17)<br>Subscriptions (n.2500)<br><br>Like Ratio: 0,09<br>View Ratio: 273,55<br><br>**VPI:** 0,25	Information flow (2)<br>Information accuracy (3)<br>Quality (3)<br>Sensitivity (3)<br><br>**VIQI:** 11	Definition (0)<br>Etiology (0)<br>Diagnosis (0)<br>Prevention (0)<br>Treatment (any) (1)<br><br>**Content score:** 1	**Educational value (GQS):** 3- Suboptimal flow, some information covered but important topics missing; somewhat useful to patients
Link:<br>https://www.youtube.com/watch?app=desktop&v=Y7NEZuIY02Q<br><br>Lenght (min):5:58<br><br>Time elapsed since upload: 1989<br><br>Target audience:<br>Both	Source: Other<br><br>Category:<br>Education<br><br>**Video Source Reliability (JAMA):** 2	Views (n.769)<br>Likes (n.0)<br>Dislikes (n.0)<br>Comments (n.0)<br>Subscriptions (n.1460)<br><br>Like Ratio: 0<br>View Ratio: 38,67<br><br>**VPI:** 0	Information flow (2)<br>Information accuracy (2)<br>Quality (3)<br>Sensitivity (3)<br><br>**VIQI:** 10	Definition (0)<br>Etiology (0)<br>Diagnosis (0)<br>Prevention (0)<br>Treatment (any) (1)<br><br>**Content score:** 1	**Educational value (GQS):** 2- Poor quality but some information present; of very limited use to patients

Link: https://www.youtube.com/watch?app=desktop&v=zhuLNJd_uLE<br><br>Lenght (min):5:00<br><br>Time elapsed since upload: 548<br><br>Target audience: Both	Source: Commercial<br><br>Category:<br>Science and technology<br><br>**Video Source Reliability (JAMA):** 2	Views (n.279)<br>Likes (n.6)<br>Dislikes (n.0)<br>Comments (n.0)<br>Subscriptions (n.6030)<br><br>Like Ratio: 2,15<br>View Ratio: 50,91<br><br>**VPI:** 1,09	Information flow (3)<br>Information accuracy (3)<br>Quality (4)<br>Sensitivity (4)<br><br>**VIQI:** 14	Definition (0)<br>Etiology (1)<br>Diagnosis (1)<br>Prevention (0)<br>Treatment (any) (1)<br><br>**Content score:** 3	**Educational value (GQS):** 3- Suboptimal flow, some information covered but important topics missing; somewhat useful to patients
Link: https://www.youtube.com/watch?app=desktop&v=i3YtaDVMsVY<br><br>Lenght (min):5:10<br><br>Time elapsed since upload (days): 2500<br><br>Target audience: Both	Source:<br>Other<br><br>Category: Science and technology<br><br>**Video Source Reliability (JAMA):** 3	Views (n.796)<br>Likes (n.0)<br>Dislikes (n.0)<br>Comments (n.0)<br>Subscriptions (n.132)<br><br>Like Ratio: 0<br>View Ratio: 31,84<br><br>**VPI:** 0	Information flow (4)<br>Information accuracy (3)<br>Quality (3)<br>Sensitivity (2)<br><br>**VIQI:** 12	Definition (1)<br>Etiology (0)<br>Diagnosis (0)<br>Prevention (1)<br>Treatment (any) (0)<br><br>**Content score:** 2	**Educational value (GQS):** 2- Poor quality but some information present; of very limited use to patients

Link<br>https://www.youtube.com/watch?app=desktop&v=vCrT3GLjrT4<br><br>Lenght (min):4:59<br><br>Time elapsed since upload (day): 1857<br><br>Target audience: Both	Source:<br>Commercial<br><br>Category:<br>Science and technology<br><br>**Video Source Reliability (JAMA):** 2	Views (n.235)<br>Likes (n.1)<br>Dislikes (n.0)<br>Comments (n.0)<br>Subscriptions (n.1440)<br><br>Like Ratio: 0<br>View Ratio: 12,6<br><br>**VPI:** 0	Information flow (2)<br>Information accuracy (2)<br>Quality (3)<br>Sensitivity (1)<br><br>**VIQI:** 8	Definition (0)<br>Etiology (0)<br>Diagnosis (0)<br>Prevention (0)<br>Treatment (any) (1)<br><br>**Content score:** 1	**Educational value (GQS):** 2- Poor quality but some information present; of very limited use to patients
Link<br>https://www.youtube.com/watch?app=desktop&v=Vvb1phusxZI<br><br>Lenght (min):5:38<br><br>Time elapsed since upload (days): 1545<br><br>Target audience: Both	Source:<br>Commercial<br><br>Category:<br>People and blogs<br><br>**Video Source Reliability (JAMA):** 4	Views (n.844)<br>Likes (n.0)<br>Dislikes (n.0)<br>Comments (n.0)<br>Subscriptions (n.6620)<br><br>Like Ratio: 0<br>View Ratio: 54,62<br><br>**VPI:** 0	Information flow (2)<br>Information accuracy (2)<br>Quality (3)<br>Sensitivity (5)<br><br>**VIQI:** 12	Definition (0)<br>Etiology (1)<br>Diagnosis (0)<br>Prevention (1)<br>Treatment (any) (1)<br><br>**Content score:** 3	**Educational value (GQS):** 2- Poor quality but some information present; of very limited use to patients

Link https://www.youtube.com/watch?app=desktop&v=6TpwWy-ZIAM<br><br>Lenght (min):8:20<br><br>Time elapsed since upload (days): 1625<br><br>Target audience: Professional	Source: Commercial<br><br>Category: Science and technology<br><br>**Video Source Reliability (JAMA):** 4	Views (n.2323)<br>Likes (n.5)<br>Dislikes (n.0)<br>Comments (n.2)<br>Subscriptions (n.908)<br><br>Like Ratio: 0,215<br>View Ratio: 142,95<br><br>**VPI:** 0,30	Information flow (2)<br>Information accuracy (2)<br>Quality (3)<br>Sensitivity (4)<br><br>**VIQI:** 11	Definition (0)<br>Etiology (1)<br>Diagnosis (0)<br>Prevention (0)<br>Treatment (any) (1)<br><br>**Content score:** 2	**Educational value (GQS):** 2- Poor quality but some information present; of very limited use to patients
Link https://www.youtube.com/watch?app=desktop&v=j74lJSlpLto<br><br>Video lenght(min) : 4:23<br><br>Time elapsed since upload (days): 2842<br><br>Target audience: Professional	Source: Other<br><br>Category: Science and technology<br><br>**Video Source Reliability (JAMA):** 3	Views (n.409)<br>Likes (n.3)<br>Dislikes (n.0)<br>Comments (n.0)<br>Subscriptions (n.7280)<br><br>Like Ratio: 0,73<br>View Ratio: 14,39<br><br>**VPI:** 10,54	Information flow (2)<br>Information accuracy (2)<br>Quality (1)<br>Sensitivity (2)<br><br>**VIQI:** 7	Definition (0)<br>Etiology (1)<br>Diagnosis (0)<br>Prevention (0)<br>Treatment (any) (0)<br><br>**Content score:** 1	**Educational value (GQS):** 2- Poor quality but some information present; of very limited use to patients

Link: https://www.youtube.com/watch?app=desktop&v=dgpaHBparHI<br><br>Lenght (min): 12:06<br><br>Time elapsed since upload (days): 1479<br><br>Target audience: Layperson	Source: Other<br><br>Category: People and blogs<br><br>**Video Source Reliability (JAMA):** 1	Views (n.2351)<br>Likes (n.0)<br>Dislikes (n.0)<br>Comments (n.48)<br>Subscriptions (n.3260)<br><br>Like Ratio: 0<br>View Ratio: 158,958<br><br>**VPI:** 0	Information flow (1)<br>Information accuracy (1)<br>Quality (1)<br>Sensitivity (1)<br><br>**VIQI:** 3	Definition (0)<br>Etiology (1)<br>Diagnosis (0)<br>Prevention (1)<br>Treatment (any) (0)<br><br>**Content score:** 2	**Educational value (GQS):** 2- Poor quality but some information present; of very limited use to patients
Link: https://www.youtube.com/watch?app=desktop&v=E93QZkvyz_g<br><br>Lenght (min): 9:32<br><br>Time elapsed since upload (days): 1511<br><br>Target audience: Professional	Source: Dentist/specialist<br><br>Category: Science and technology<br><br>**Video Source Reliability (JAMA):** 2	Views (n.56)<br>Likes (n.2)<br>Dislikes (n.0)<br>Comments (n.0)<br>Subscriptions (n.192)<br><br>Like Ratio: 3,57<br>View Ratio: 3,7<br><br>**VPI:** 0,132	Information flow (3)<br>Information accuracy (4)<br>Quality (4)<br>Sensitivity (3)<br><br>**VIQI:** 14	Definition (0)<br>Etiology (0)<br>Diagnosis (0)<br>Prevention (0)<br>Treatment (any) (1)<br><br>**Content score:** 1	**Educational value (GQS):** 2- Poor quality but some information present; of very limited use to patients

Link: https://www.youtube.com/watch?app=desktop&v=BUDMkFgucG4<br><br>Lenght (min): 4:07<br><br>Time elapsed since upload (days): 1181<br><br>Target audience: Professional	Source: Commercial<br><br>Category: Education<br><br>**Video Source Reliability (JAMA):** 3	Views (n.158)<br>Likes (n.2)<br>Dislikes (n.0)<br>Comments (n.0)<br>Subscriptions (n.1090)<br><br>Like Ratio: 1,26<br>View Ratio: 13,37<br><br>**VPI:** 16,85	Information flow (1)<br>Information accuracy (1)<br>Quality (1)<br>Sensitivity (1)<br><br>**VIQI:** 4	Definition (0)<br>Etiology (0)<br>Diagnosis (0)<br>Prevention (0)<br>Treatment (any) (1)<br><br>**Content score:** 1	**Educational value (GQS):** 1- Poor quality; very unlikely to be of any use to patients
Link: New Approaches for peri-implant soft tissue boosting - Dr. Giorgio Tabanella - YouTube<br><br>Lenght (min):16:45<br><br>Time elapsed since upload (days): 804<br><br>Target audience: Professional	Source: Dentist/specialist<br><br>Category: Education<br><br>**Video Source Reliability (JAMA):** 3	Views (n.481)<br>Likes (n.9)<br>Dislikes (n.0)<br>Comments (n.0)<br>Subscriptions (n.4889<br><br>Like Ratio: 1,87<br>View Ratio: 59,60<br><br>**VPI:** 1,11	Information flow (4)<br>Information accuracy (4)<br>Quality (4)<br>Sensitivity (3)<br><br>**VIQI:** 15	Definition (1)<br>Etiology (0)<br>Diagnosis (1)<br>Prevention (0)<br>Treatment (any) (1)<br><br>**Content score:** 3	**Educational value (GQS):** 3- Suboptimal flow, some information covered but important topics missing; somewhat useful to patients

Link:<br>Reverse Torque Removal of a Failing and Infected Dental Implant - YouTube<br><br>Lenght (min):4:57<br><br>Time elapsed since upload (days): 45<br><br>Target audience: Both	Source:<br>Dentist/specialist<br><br>Category:<br>Education<br><br>**Video Source Reliability (JAMA):** 2	Views (n.106)<br>Likes (n.1)<br>Dislikes (n.0)<br>Comments (n.0)<br>Subscriptions (n.3890)<br><br>Like Ratio: 0,94<br>View Ratio: 235,55<br><br>**VPI:** 2,214	Information flow (4)<br>Information accuracy (4)<br>Quality (4)<br>Sensitivity (3)<br><br>**VIQI:** 15	Definition (1)<br>Etiology (0)<br>Diagnosis (0)<br>Prevention (1)<br>Treatment (any) (1)<br><br>**Content score:** 3	**Educational value (GQS):** 4- Good quality and flow, most important topics covered; useful to patients
Link:<br>Lecture on “No Peri-implantitis” by Dr Chandrahas Bathini - YouTube<br><br>Lenght (min):5:03<br><br>Time elapsed since upload (days): 291<br><br>Target audience: Professional	Source:<br>Dentist/specialist<br>Category:<br>Education<br><br>**Video Source Reliability (JAMA):** 2	Views (n.24)<br>Likes (n.1)<br>Dislikes (n.0)<br>Comments (n.0)<br>Subscriptions (n.5440)<br><br>Like Ratio: 4,17<br>View Ratio: 8,25<br><br>**VPI:** 0,34	Information flow (2)<br>Information accuracy (2)<br>Quality (1)<br>Sensitivity (1)<br><br>**VIQI:** 6	Definition (1)<br>Etiology (0)<br>Diagnosis (0)<br>Prevention (0)<br>Treatment (any) (1)<br><br>**Content score:** 2	**Educational value (GQS):** 1- Poor quality; very unlikely to be of any use to patients

Link: https://www.youtube.com/watch?v=afOjNTOoPEE<br><br>Lenght (min):11:03<br><br>Time elapsed since upload (days): 2368<br><br>Target audience: Both	Source: Dentist/specialist<br><br>Category: People and blogs<br><br>**Video Source Reliability (JAMA):** 3	Views (n.33823)<br>Likes (n.273)<br>Dislikes (n.0)<br>Comments (n.16)<br>Subscriptions (n.3460)<br><br>Like Ratio: 0,81<br>View Ratio: 1428,33<br><br>**VPI:** 11,57	Information flow (5)<br>Information accuracy (5)<br>Quality (5)<br>Sensitivity (3)<br><br>**VIQI:** 18	Definition (1)<br>Etiology (0)<br>Diagnosis (1)<br>Prevention (0)<br>Treatment (any) (1)<br><br>**Content score:** 3	**Educational value (GQS):** 4- Good quality and flow, most important topics covered; useful to patients
Link: https://www.youtube.com/watch?app=desktop&v=DtBmhz6ja7A<br><br>Lenght (min):4:59<br><br>Time elapsed since upload (days): 1168<br><br>Target audience: Professional	Source: Dentist/specialist Commercial<br><br>Category: Science and technology<br><br>**Video Source Reliability (JAMA):** 4	Views (n.586)<br>Likes (n.7)<br>Dislikes (n.0)<br>Comments (n.0)<br>Subscriptions (n.908)<br><br>Like Ratio: 1,19<br>View Ratio: 50,17<br><br>**VPI:** 0,60	Information flow (3)<br>Information accuracy (3)<br>Quality (3)<br>Sensitivity (5)<br><br>**VIQI:** 14	Definition (0)<br>Etiology (0)<br>Diagnosis (1)<br>Prevention (0)<br>Treatment (any) (1)<br><br>**Content score:** 2	**Educational value (GQS):** 3- Suboptimal flow, some information covered but important topics missing; somewhat useful to patients

Link:<br>https://www.youtube.com/watch?app=desktop&v=5lrrm-ra5FY<br><br>Lenght (min):10:48<br><br>Time elapsed since upload (days): 798<br><br>Target audience: Professional	Source:<br>Commercial<br><br>Category:<br>Science and technology<br><br>**Video Source Reliability (JAMA):** 1	Views (n.239)<br>Likes (n.8)<br>Dislikes (n.0)<br>Comments (n.0)<br>Subscriptions (n.953)<br><br>Like Ratio: 3,35<br>View Ratio: 29,95<br><br>**VPI:** 1,00	Information flow (2)<br>Information accuracy (3)<br>Quality (3)<br>Sensitivity (2)<br><br>**VIQI:** 10	Definition (0)<br>Etiology (0)<br>Diagnosis (0)<br>Prevention (0)<br>Treatment (any) (1)<br><br>**Content score:** 1	**Educational value (GQS):** 2- Poor quality but some information present; of very limited use to patients
Link:<br>https://www.youtube.com/watch?v=uiu6GgB8bZk<br><br>Lenght (min):15:34<br><br>Time elapsed since upload (days): 164<br><br>Target audience:<br>Professional	Source:<br>Hospital/university/scientific dental associations<br><br>Category:<br>People and blogs<br><br>**Video Source Reliability (JAMA):** 1	Views (n.608)<br>Likes (n.12)<br>Dislikes (n.0)<br>Comments (n.0)<br>Subscriptions (n.953)<br><br>Like Ratio: 1,97<br>View Ratio: 370,73<br><br>**VPI:** 7,30	Information flow (3)<br>Information accuracy (4)<br>Quality (2)<br>Sensitivity (4)<br><br>**VIQI:** 13	Definition (1)<br>Etiology (1)<br>Diagnosis (0)<br>Prevention (0)<br>Treatment (any) (1)<br><br>**Content score:** 3	**Educational value (GQS):** 2- Poor quality but some information present; of very limited use to patients

Link: https://www.youtube.com/watch?v=n6knCNQrips<br><br>Lenght (min):15:44<br><br>Time elapsed since upload (days): 1031<br><br>Target audience: Both	Source: Hospital/university/scientific dental associations<br><br>Category: Film and animation<br><br>**Video Source Reliability (JAMA):** 4	Views (n.27194)<br>Likes (n.333)<br>Dislikes (n.0)<br>Comments (n.27)<br>Subscriptions (n.31600)<br><br>Like Ratio: 1,22<br>View Ratio: 2637,63<br><br>**VPI:** 32,18	Information flow (5)<br>Information accuracy (5)<br>Quality (5)<br>Sensitivity (5)<br><br>**VIQI:** 20	Definition (1)<br>Etiology (1)<br>Diagnosis (1)<br>Prevention (1)<br>Treatment (any) (1)<br><br>**Content score:** 5	**Educational value (GQS):** 5- Excellent quality and flow; highly useful to patients
Link: https://www.youtube.com/watch?v=_CsR64ElBoQ<br><br>Lenght (min):4:14<br><br>Time elapsed since upload (days): 2516<br><br>Target audience: Professional	Source: Commercial<br><br>Category: Science and technology<br><br>**Video Source Reliability (JAMA):** 2	Views (n.5453)<br>Likes (n.18)<br>Dislikes (n.0)<br>Comments (n.0)<br>Subscriptions (n.33100)<br><br>Like Ratio: 0,33<br>View Ratio: 216,73<br><br>**VPI:** 0,71	Information flow (2)<br>Information accuracy (3)<br>Quality (2)<br>Sensitivity (3)<br><br>**VIQI:** 10	Definition (1)<br>Etiology (0)<br>Diagnosis (0)<br>Prevention (0)<br>Treatment (any) (1)<br><br>**Content score:** 2	**Educational value (GQS):** 2- Poor quality but some information present; of very limited use to patients

Link: https://www.youtube.com/watch?v=X8cmzaIMNFw<br><br>Lenght (min):4:05<br><br>Time elapsed since upload (days): 1612<br><br>Target audience: Professional	Source: Commercial<br><br>Category: Science and technology<br><br>**Video Source Reliability (JAMA):** 2	Views (n.600)<br>Likes (n.5)<br>Dislikes (n.0)<br>Comments (n.0)<br>Subscriptions (n.4530)<br><br>Like Ratio: 0,83<br>View Ratio: 37,22<br><br>**VPI:** 0,31	Information flow (3)<br>Information accuracy (2)<br>Quality (3)<br>Sensitivity (3)<br><br>**VIQI:** 11	Definition (0)<br>Etiology (0)<br>Diagnosis (0)<br>Prevention (0)<br>Treatment (any) (1)<br><br>**Content score:** 1	**Educational value (GQS):** 1- Poor quality; very unlikely to be of any use to patients
Link: https://www.youtube.com/watch?v=M361YtdeoiM<br><br>Lenght (min):15:21<br><br>Time elapsed since upload (days): 769<br><br>Target audience: Both	Source: Dentist/specialist<br><br>Category: Education<br><br>**Video Source Reliability (JAMA):** 3	Views (n.1791)<br>Likes (n.38)<br>Dislikes (n.0)<br>Comments (n.0)<br>Subscriptions (n.6030)<br><br>Like Ratio: 2,12<br>View Ratio: 232,90<br><br>**VPI:** 4,94	Information flow (4)<br>Information accuracy (4)<br>Quality (5)<br>Sensitivity (5)<br><br>**VIQI:** 18	Definition (1)<br>Etiology (1)<br>Diagnosis (0)<br>Prevention (0)<br>Treatment (any) (1)<br><br>**Content score:** 4	**Educational value (GQS):** 3- Suboptimal flow, some information covered but important topics missing; somewhat useful to patients

Link: https://www.youtube.com/watch?v=hiubSWcZLCM<br><br>Lenght (min):4:54<br><br>Time elapsed since upload (days): 975<br><br>Target audience: Professional	Source: Hospital/university/scientific dental associations<br><br>Category: Education<br><br>**Video Source Reliability (JAMA):** 3	Views (n.3543)<br>Likes (n.35)<br>Dislikes (n.0)<br>Comments (n.1)<br>Subscriptions (n.7280)<br><br>Like Ratio: 0,99<br>View Ratio: 363,38<br><br>**VPI:** 3,60	Information flow (4)<br>Information accuracy (4)<br>Quality (2)<br>Sensitivity (4)<br><br>**VIQI:** 14	Definition (1)<br>Etiology (1)<br>Diagnosis (1)<br>Prevention (1)<br>Treatment (any) (1)<br><br>**Content score:** 5	**Educational value (GQS):** 3- Suboptimal flow, some information covered but important topics missing; somewhat useful to patients
Link: https://www.youtube.com/watch?v=qh959xoQJck<br><br>Lenght (min):7:48<br><br>Time elapsed since upload (days): 554<br><br>Target audience: Both	Source: Dentist/specialist<br><br>Category: People and blogs<br><br>**Video Source Reliability (JAMA):** 4	Views (n.471)<br>Likes (n.5)<br>Dislikes (n.0)<br>Comments (n.0)<br>Subscriptions (n.4440)<br><br>Like Ratio: 1,06<br>View Ratio: 85,01<br><br>**VPI:** 0,90	Information flow (3)<br>Information accuracy (3)<br>Quality (1)<br>Sensitivity (2)<br><br>**VIQI:** 9	Definition (1)<br>Etiology (0)<br>Diagnosis (1)<br>Prevention (0)<br>Treatment (any) (1)<br><br>**Content score:** 3	**Educational value (GQS):** 2- Poor quality but some information present; of very limited use to patients

Link: https://www.youtube.com/watch?v=uvb5udFZ3eA<br><br>Lenght (min):4:50<br><br>Time elapsed since upload (days): 973<br><br>Target audience: Professionals	Source: Dentist/specialist<br><br>Category: education<br><br>**Video Source Reliability (JAMA):** 3	Views (n.1791)<br>Likes (n.53)<br>Dislikes (n.0)<br>Comments (n.4)<br>Subscriptions (n.7430)<br><br>Like Ratio: 2,96<br>View Ratio: 184,07<br><br>**VPI:** 5,45	Information flow (3)<br>Information accuracy (3)<br>Quality (1)<br>Sensitivity (4)<br><br>**VIQI:** 11	Definition (0)<br>Etiology (0)<br>Diagnosis (0)<br>Prevention (1)<br>Treatment (1)<br><br>**Content score:** 2	**Educational value (GQS):** 3- Suboptimal flow, some information covered but important topics missing; somewhat useful to patient
Link: https://www.youtube.com/watch?v=5lrrm-ra5FY<br><br>Lenght (min):10:48<br><br>Time elapsed since upload (days): 802<br><br>Target audience: Professionals	Source: Dentist/specialist<br><br>Category: Science and technology<br><br>**Video Source Reliability (JAMA):** 2	Views (n.241)<br>Likes (n.8)<br>Dislikes (n.0)<br>Comments (n.0)<br>Subscriptions (n.953)<br><br>Like Ratio: 3,32<br>View Ratio: 30,05<br><br>**VPI:** 1,00	Information flow (2)<br>Information accuracy (2)<br>Quality (3)<br>Sensitivity (2)<br><br>**Video Information and Quality Index (VIQI):** 9	Definition (0)<br>Etiology (0)<br>Diagnosis (0)<br>Prevention (0)<br>Treatment (1)<br><br>**Content score:** 1	**Educational value (GQS):** 2- Poor quality but some information present; of very limited use to patients

Link: https://www.youtube.com/watch?v=Vdl-fF7_vvU<br><br>Video lenght(min): 19:19<br><br>Time elapsed since upload (days): 27<br><br>Target audience: Professionals	Source: Dentist/specialist<br><br>Category: Education<br><br>**Video Source Reliability (JAMA):** 3	Views (n.910)<br>Likes (n.49)<br>Dislikes (n.0)<br>Comments (n.6)<br>Subscriptions (n.7430)<br><br>Like Ratio: 5,38<br>View Ratio: 3370,37<br><br>**VPI:** 181,33	Information flow (4)<br>Information accuracy (5)<br>Quality (4)<br>Sensitivity (4)<br><br>**Video Information and Quality Index (VIQI):** 17	Definition (1)<br>Etiology (1)<br>Diagnosis (0)<br>Prevention (1)<br>Treatment (0)<br><br>**Content score:** 3	**Educational value (GQS):** 3- Suboptimal flow, some information covered but important topics missing; somewhat useful to patient
Link: https://www.youtube.com/watch?v=AuqDqYwyTu8<br><br>Lenght (min):4:33<br><br>Time elapsed since upload (days): 649<br><br>Target audience: Professionals	Source: Dentist/specialist<br><br>Category: Education<br><br>**Video Source Reliability (JAMA):** 2	Views (n.2221)<br>Likes (n.67)<br>Dislikes (n.0)<br>Comments (n.6)<br>Subscriptions (n.7430)<br><br>Like Ratio: 3,02<br>View Ratio: 342,22<br><br>**VPI:** 10,33	Information flow (5)<br>Information accuracy (5)<br>Quality (2)<br>Sensitivity (4)<br><br>**Video Information and Quality Index (VIQI):** 16	Definition (0)<br>Etiology (0)<br>Diagnosis (0)<br>Prevention (0)<br>Treatment (y/)<br><br>**Content score:** 4	**Educational value (GQS):** 4- Good quality and flow, most important topics covered; useful to patients

Link: https://www.youtube.com/watch?v=zhuLNJd_uLE<br><br>Lenght (min):5:00<br><br>Time elapsed since upload (days): 552<br><br>Target audience: Both	Source: Dentist/specialist<br><br>Category: Film and animation<br><br>**Video Source Reliability (JAMA):** 2	Views (n.283)<br>Likes (n.6)<br>Dislikes (n.0)<br>Comments (n.0)<br>Subscriptions (n.6030)<br><br>Like Ratio: 2,12<br>View Ratio: 51,27<br><br>**VPI:** 1,087	Information flow (3)<br>Information accuracy (3)<br>Quality (4)<br>Sensitivity (3)<br><br>**VIQI:** 13	Definition (1)<br>Etiology (0)<br>Diagnosis (1)<br>Prevention (0)<br>Treatment (any) (1)<br><br>**Content score:** 3	**Educational value (GQS):** 3- Suboptimal flow, some information covered but important topics missing; somewhat useful to patients
Link: https://www.youtube.com/watch?v=AW5loeSa2wM<br><br>Lenght (min):5:11<br><br>Time elapsed since upload (days): 1776<br><br>Target audience: Professional	Source: Dentist/specialist<br><br>Category: Education<br><br>**Video Source Reliability (JAMA):** 2	Views (n.375)<br>Likes (n.4)<br>Dislikes (n.0)<br>Comments (n.0)<br>Subscriptions (n.691)<br><br>Like Ratio: 1,07<br>View Ratio: 21,11<br><br>**VPI:** 0,22	Information flow (3)<br>Information accuracy (3)<br>Quality (4)<br>Sensitivity (3)<br><br>**Video Information and Quality Index (VIQI):** 13	Definition (0)<br>Etiology (0)<br>Diagnosis (0)<br>Prevention (1)<br>Treatment (any) (1)<br><br>**Content score:** 2	**Educational value (GQS):** 2- Poor quality information but some present; of very limited use to patients

Link: https://www.youtube.com/watch?v=62lqwNAs1Tw<br>Lenght (min):9:08<br>Time elapsed since upload (days): 1973<br>Target audience: Both	Source: Commercial<br>Category: Science and technology<br>**Video Source Reliability (JAMA):** 2	Views (n.3272)<br>Likes (n.5)<br>Dislikes (n.0)<br>Comments (n.0)<br>Subscriptions (n.9)<br>Like Ratio: 0,15<br>View Ratio: 165,84<br>**VPI:** 0,25	Information flow (2)<br>Information accuracy (2)<br>Quality (3)<br>Sensitivity (3)<br>**VIQI:** 10	Definition (0)<br>Etiology (0)<br>Diagnosis (0)<br>Prevention (1)<br>Treatment (any) (1)<br>**Content score:** 2	**Educational value (GQS):** 3- Suboptimal flow, some informationcovered but important topics missing; somewhat useful to patients
Link: https://www.youtube.com/watch?v=z3JXpjibrjw<br>Lenght (min):4:10<br>Time elapsed since upload (days): 1970<br>Target audience: Both	Source: Dentist/specialist<br>Category: People and blogs<br>**Video Source Reliability (JAMA):** 2	Views (n.449)<br>Likes (n.0)<br>Dislikes (n.0)<br>Comments (n.0)<br>Subscriptions (n.1460)<br>Like Ratio: 0<br>View Ratio: 22,79<br>**VPI:** 0	Information flow (2)<br>Information accuracy (2)<br>Quality (1)<br>Sensitivity (3)<br>**VIQI:** 8	Definition (0)<br>Etiology (0)<br>Diagnosis (0)<br>Prevention (1)<br>Treatment (any) (y/)<br>**Content score:** 2	**Educational value (GQS):** 2- Poor quality but some information present; of very limited use to patients

Link: https://www.youtube.com/watch?v=SBc1TGtJXJM<br><br>Lenght (min):4:56<br><br>Time elapsed since upload (days): 296<br><br>Target audience: Both	Source: Dentist/specialist<br><br>Category: Film and animation<br><br>**Video Source Reliability (JAMA):** 3	Views (n.760)<br>Likes (n.12)<br>Dislikes (n.0)<br>Comments (n.2)<br>Subscriptions (n.7200)<br><br>Like Ratio: 1,58<br>View Ratio: 256,76<br><br>**VPI:** 4,06	Information flow (4)<br>Information accuracy (4)<br>Quality (5)<br>Sensitivity (4)<br><br>**VIQI:** 17	Definition (1)<br>Etiology (0)<br>Diagnosis (0)<br>Prevention (0)<br>Treatment (any) (y/)<br><br>**Content score:** 4	**Educational value (GQS): 4-** Good quality and flow, most important topics covered
Link: https://www.youtube.com/watch?v=Y7NEZuIY02Q<br><br>Video lenght(min): 5:58<br><br>Time elapsed since upload (days): 1994<br><br>Target audience: Layperson	Source: Dentist/specialist<br><br>Category: Science and technology<br><br>**Video Source Reliability (JAMA):** 2	Views (n.773)<br>Likes (n.0)<br>Dislikes (n.0)<br>Comments (n.0)<br>Subscriptions (n.1460)<br><br>Like Ratio: 0<br>View Ratio: 38,77<br><br>**VPI:** 0	Information flow (3)<br>Information accuracy (2)<br>Quality (3)<br>Sensitivity (4)<br><br>**VIQI:** 12	Definition (0)<br>Etiology (0)<br>Diagnosis (0)<br>Prevention (0)<br>Treatment (any) (1)<br><br>**Content score:** 2	**Educational value (GQS):** 2- Poor quality but some information present; of very limited use to patients

Link: https://www.youtube.com/watch?v=1M0eHWtJDLE<br><br>Video lenght(min): 12:39<br><br>Time elapsed since upload (days): 448<br><br>Target audience: Layperson	Source: Dentist/specialist<br><br>Category: Education<br><br>**Video Source Reliability (JAMA):** 3	Views (n.99)<br>Likes (n.2)<br>Dislikes (n.0)<br>Comments (n.0)<br>Subscriptions (n.421)<br><br>Like Ratio: 2,02<br>View Ratio: 22,10<br><br>**VPI: :** 44,64	Information flow (3)<br>Information accuracy (4)<br>Quality (3)<br>Sensitivity (3)<br><br>**VIQI:** 13	Definition (1)<br>Etiology (1)<br>Diagnosis (1)<br>Prevention (0)<br>Treatment (0)<br><br>**Content score:** 3	**Educational value (GQS):** 3- Suboptimal flow, some information covered but important topics missing; somewhat useful to patients
